# Biofilm inhibition of denture cleaning tablets and carvacrol on denture bases produced with different techniques

**DOI:** 10.1007/s00784-024-05810-3

**Published:** 2024-07-05

**Authors:** Zeynep Sahin, Nazire Esra Ozer, Abdulhamit Calı

**Affiliations:** 1 Department of Prosthodontics, Faculty of Dentistry, Lokman Hekim University, Söğütözü. 2179 St., Çankaya, Ankara, 06510 Turkey; 2 Vocational School of Health Services, Medical Laboratory Techniques Program, Lokman Hekim University, Ankara, Turkey

**Keywords:** Antibiofilm, CAD/CAM, Carvacrol, 3D printing, Denture cleansers

## Abstract

**Objectives:**

This study compares the biofilm inhibition effects of denture cleaning tablets, carvacrol, and their combined use against *Candida albicans* on denture bases produced with different techniques. Additionally, the surface roughness and contact angles of these denture bases were evaluated.

**Materials and methods:**

Test samples were prepared from four different denture base materials (cold-polymerized, heat-polymerized, CAD/CAM milling, and 3D-printed). The surface roughness and contact angles of the test samples were measured using a profilometer and goniometer, respectively. For the evaluation of biofilm inhibition, samples were divided into 5 subgroups: Corega and carvacrol, separately and combined treatments, positive (inoculated with *C. albicans*) and negative control (non-inoculated with *C. albicans*, only medium). Biofilm mass was determined using the crystal violet method. An additional prepared test sample for each subgroup was examined under scanning electron microscopy (SEM).

**Results:**

The surface roughness values of the 3D-printed test samples were found to be statistically higher than the other groups (*P* < .001). The water contact angle of all test materials was not statistically different from each other (*P* > .001). Corega and carvacrol, separately and combined, significantly decreased the amount of biofilm on all surfaces (*P* < .0001). Treatment of corega alone and in combination with carvacrol to the 3D-printed material caused less *C. albicans* inhibition than the other groups (*P* < .001; *P* < .05).

**Conclusions:**

The surface roughness values of all test groups were within the clinically acceptable threshold. Although Corega and carvacrol inhibited *C. albicans* biofilms, their combined use did not show a synergistic effect.

**Clinical relevance:**

Carvacrol may be used as one of the disinfectant agents for denture cleaning due to its biofilm inhibition property.

## Introduction

Complete and overdenture prostheses have long been recognized as effective treatment options for individuals with complete edentulism [1]. Poly (methyl methacrylate) (PMMA), is one of the substances that are most frequently employed in the creation of these prostheses [2]. The denture base is traditionally prepared using either heat or cold curing [3]. The benefits of employing traditional PMMA resins are their simplicity in handling and manipulation, biocompatibility, acceptable aesthetics, stability, and affordability [4]. PMMA does have certain drawbacks, though, including polymerization shrinkage, poor strength, limited wear resistance, and, most importantly high susceptibility to microbial and fungal colonization [2]. Following the innovation in digital dentistry and the development of new materials, numerous clinical, laboratory, and biomaterial processes have been developed for the production of digital complete dentures [1]. These prostheses can be designed and manufactured using computer-aided design and manufacturing (CAD/CAM) methods that either use additive (3D printing) or subtractive (milling) manufacturing processes [3, 4]. CAD/CAM production methods have reduced treatment times and improved clinical reproducibility [5].

Denture stomatitis (DS) can affect 15–70% of people who wear dentures [3]. DS, a multifactorial oral pathological condition, is triggered by the accumulation of biofilm on both the tissues and the surface of the denture, and it manifests with varying degrees of erythema, bleeding, and halitosis [4]. Poor oral hygiene, trauma, and *Candida albicans (C. albicans)* infections are the main etiological factors for DS [4].

Compared to smooth surfaces, rough surfaces are more prone to bacterial adherence and plaque buildup [6]. The manufacturing process used affects the surface topography of the material, which is formed as surface defects, irregularities, cracks, and porosities [1]. The prosthesis may contain such as microscopic defects that result in biofilm and microbe harborage [7].

Oral candidiasis is prevented in various ways, some of which include cleaning dentures chemically, mechanically, or with a combination of the two [6]. Mechanical cleaning is accomplished with a toothbrush and an ultrasonic cleaner [8]. With the chemical cleaning procedure, dentures must be submerged in a range of active ingredients, including enzymes, hypochlorite solutions, disinfectants, alkaline peroxides, and diluted organic and inorganic acids [6]. However, these products can degrade acrylic resin, altering its characteristics such as color, roughness, hardness, and flexural strength, decreasing the longevity of dental prostheses [9]. Natural products may be another option for synthetic chemicals for cleaning prostheses [10]. Carvacrol (2-methyl-5-(1-methylethyl) phenol) (C_10_H_14_O) is a substance found in the essential oils (EOs) of many aromatic plants, including thyme and oregano (Origanum sp.), which are also aromatic [11]. According to the Food and Drug Administration (FDA), carvacrol is a substance that is generally recognized as safe (GRAS) [12]. Carvacrol has been added to the soft liner material used in denture relining processes because of its antibacterial and antibiofilm properties [11]. EOs are often only used in dentistry as an antiseptic on patients with dentate conditions. It is available as mouthwash, consisting of a blend of several substances derived from various plant species [13]. EO helps those who wear dentures have less stomatitis [14, 15].

Since DS is difficult to treat, efforts are being made to prevent it [3]. In some studies, antimicrobial nanoparticles were added to the prosthesis base resin [2, 16]. This may alter the material’s physical characteristics, and it is unknown how long-term effective they will be. Other studies attempted to prevent microbial adhesion by using coating material [17, 18]. There is a possibility of wear on this material over time. As a result, the search continues for improved fabrication techniques and materials [3]. Additionally, the disinfectant protocol to be applied to prosthetic bases with different produced methods is important to prevent biofilm.

Studies on the surface properties and *C. albicans* adhesion of denture bases produced by traditional and digital methods were conducted [1, 3]. Although the effects of denture cleaning tablets and agents on the surface properties and microbial retention of digital dentures have been examined in one study [4], no study has yet been found that examines the antibiofilm properties of denture cleaning tablets, carvacrol, and the combined use of these two materials (whether they show synergistic effects) against *C. albicans* on digital and traditional denture bases.

This study aimed to compare the biofilm inhibition effects of denture cleaning tablets, carvacrol, and combined use of *C. albicans* on denture bases produced by digital and conventional methods. Additionally, the surface properties and contact angles of the denture base materials were evaluated.

The null hypotheses of this study are: (1) denture cleaning tablet and carvacrol alone and in combination will show similar biofilm inhibition in digitally and conventionally produced test samples, (2) combined use will not cause greater inhibition of *C.albicans* than use alone and will not show a synergistic effect, (3) different production methods will not affect the surface roughness and contact angle values.

## Materials and methods

A total of 216 test samples were prepared from four different denture base materials (cold-polymerized, heat-polymerized, CAD/CAM milling, and 3D printing) with 10 × 2 mm dimensions to evaluate surface and anti-biofilm properties. The study’s materials are listed in Table 1. The sample size was calculated based on the previous similar study [19] data to estimate the power of 0.85 at α = 0.05 (G Power 3.1.9.2). Freitas et al. [19] reported the values of *C. albicans* adhesion in four different groups (CAD/CAM milling, 3D printing, heat, and microwave polymerization). The results for each group were as follows: CAD-CAM Milled Group - GM (Log CFU/mL 3.74 ± 0.57), 3D Printed - GP (Log CFU/mL: 5.77 ± 0.36), and conventional methods (Log CFU/mL: 5.23 ± 0.48 for GCV and GCL respectively). Accordingly, the minimum number of sample groups per subgroup was determined as 7 (total sample size 28). Considering that the surface roughness value ranges give very close measurements to each other, to increase the power of the study, and possible sample loss during the experiments [20], the number n was taken as 10 for the surface roughness experiment.

**Table 1:** Materials used in the study

Denture resin	Type	Composition	Manufacturer
Meliodent	Cold Polymerizing Acrylic Resin	**Powder**: PMMA **Liquid**: MMA > %90 tetramethylene dimethacrylate %0–5 2-(2 H-Benzotriazole-2-yl)-4-methyl phenol < %1 N, N-dimethyl-p-toluidine < %1	Heraeus Kulzer GmbH, Hanau, Germany
Procryla	Heat Polymerizing Acrylic Resin	**Powder**: PMMA **Liquid**: MMA	Vertex Dental BV, Netherlands
Yamahachi	Resin Disc	PMMA 99.5%, pigments < 1.0%	Yamahachi Dental MFG, Aichi-Pref, Japan
Curo Denture	Resin for 3D Printing	Aliphatic difunctional methacrylate,2.2’ethylenedioxydiethyl dimethacrylate, Aliphatic Urethane Acrylate, Phosphine oxide	MACK4D, Ackuretta Technologies Pvt Ltd, Neukiritzsch, Germany

(3D: Three-dimensional, PMMA: polymethyl methacrylate, MMA: Methyl methacrylate)

PMMA (cold-polymerized, heat-polymerized) acrylic resin discs were made in compliance with the guidelines provided by the manufacturer. The samples were made utilizing a traditional flasking and pressure-pack method from an acrylic resin denture base material. The test samples produced by the conventional method were obtained by milling wax blocks of the same dimension to ensure standardization. The wax samples were placed in the flask. Following the removal of the wax, layers of separating material were administered. The heat-polymerized test samples were mixed according to the manufacturer’s instructions and the flask-finishing processes were carried out [4]. The heat-polymerization process was carried out in a water bath for 90 min at 74 °C, followed by 30 min at 100 °C. Similarly, in cold polymerized samples, negative molds were obtained after the wax melted. Powder and liquid were mixed following the manufacturer [3, 4]. The process was conducted for 2 min at room temperature, followed by 10 min at 55 °C under 2 bar pressure in a pressure curing pot. CAD/CAM PMMA samples were created via a computer program and subsequently milled using a milling machine (CORiTEC 350i, imes-icore GmbH). The 3D printed samples were produced in 0° orientation with a layer thickness of 0.05 mm using a 3D printer (FreeShape 120, Ackuretta). The surface of the 3D printed sample was then post-cured for three minutes under UV light (UV Box, Acuretta) after being cleaned for five minutes with isopropyl alcohol in an ultrasonic cleaner. The finishing and polishing processes of the samples were carried out using a wet sanding process from 400 grit to 1200 grit and polishing paste (Ivoclar, Vivadent) with a polishing cloth respectively.

The surface roughness of test samples (*n* = 10 per group) was measured using a profilometer (Perthometer M2, Mahr). Before measuring each test group, a reference block was used to calibrate the device. At three sites along the same vertical line, horizontal parallel measurements were made from the surface of each test sample (with a measurement length of 5.6 mm, and a speed of 0.5 mm/s). The arithmetic mean of the three measurements for each test sample was used to get the average surface roughness value of each sample [6].

Using the sessile drop method, the water contact angle of each sample was measured with a contact angle meter (OCA 15 plus, Dataphysics Instruments GmbH). 2.0 μL of distilled water was put onto the test surface once each sample had been positioned so that it was straight and not inclined. The right and left static contact angles were then measured as soon as a single water droplet touched the sample’s center on both sides. Each drop and the disk surface were photographed, and the water contact angle was recorded [19, 21].

Corega denture cleaning tablet (Stafford-Miller), carvacrol (Bldpharm), and fluconazole (Bldpharm) were commercially available and used in this study. The concentrations of stock solutions of Corega, carvacrol, and fluconazole were prepared according to EUCAST recommendations as twenty times the concentration to be studied [21]. Following the manufacturer’s recommendation, a stock solution was prepared for use in microbiological studies by dissolving Corega tablets in a 200 mL beaker containing 40 °C sterile distilled water.

*C. albicans* (ATCC 10231) strain was used in this study. After incubation in Sabouraud dextrose agar (SDA) overnight, they were passaged into Sabouraud dextrose broth (SDB) medium and incubated at 37 °C for 24 h. After incubation, the yeast suspension was prepared as 0.5 McFarland. 0.5 mL of this prepared suspension was added to 9.5 mL of medium and diluted 1/20 [22, 23].

The microdilution method was used to determine the MIC values of corega, carvacrol, and fluconazole against *C. albicans*. 90 μl of SDB was dispensed into the first well of the 96-well microplate and 50 μl each into the other nine wells. The eleventh and twelfth wells were used for growth and sterility control, respectively. 10 μl of the prepared solutions (Corega, carvacrol, and fluconazole) were added into the first well and serial dilution was performed. Then 50 μl of the prepared yeast suspension was dispensed into all wells except the last well. After incubation at 37 °C for 24 h, the lowest concentration at which no growth was determined as the MIC value [22, 24].

Biofilm formation and inhibition experiments were performed on denture materials obtained by cold-polymerized, heat-polymerized, CAD/CAM milling, and 3D printed methods (*n* = 8) using the method proposed by Christensen [25]. All samples prepared for microbiological experiments were sterilized in an autoclave (at 121 °C for 15 min). Test materials were placed in sterile glass tubes. A suspension of 0.5 McFarland was prepared in tryptic soy broth (TSB) medium containing 1% glucose from the *C. albicans* (ATCC 10231) strain produced by overnight incubation on SDA agar. From this suspension, 500 μl was dispensed into 40 of the prepared tubes. For the negative control, only TSB was added to the tubes. For *C. albicans* adhesion, they were incubated at 37 °C and 75 rpm for two hours. After incubation, the tubes were aspirated with a pipette and washed with phosphate buffer (PBS) to non-adherent cells were removed. 500 μl of TSB was again dispensed into the tubes and incubated at 37 °C and 75 rpm for 48 h [26].

Following biofilm formation, all tubes were washed with PBS and test materials were transferred to new sterile tubes. Each group of test materials was divided into five subgroups (*n* = 8). Corega alone (20 mg/ml) was added to the first group, carvacrol alone (0.128 mg/ml) was added to the second group, and Corega/carvacrol combination (20 mg/ml / 0.128 mg/ml) was added to the third group in 500 μl. For the other groups, only 500 μl of sterile distilled water was added for positive control and negative control to ensure standardization. The positive control was obtained by inoculating *C. albicans* into the tubes in which the test samples were placed. In the negative control, *C. albicans* was not inoculated in the tubes. Only the medium and samples were incubated. They were then incubated at 37 °C and 75 rpm for eight hours. The amount of adherent biofilm biomass was measured using the crystal violet staining technique [25, 26].

After eight hours, all solutions in the tubes were pipetted out and washed twice with PBS. After drying at room temperature, 95% methanol was added to the tubes and kept for 15 min. The tubes were then evacuated and dried at room temperature. Then 0.1% crystal violet was dispensed into all tubes and left for 30 min. After staining, the tubes were washed twice with PBS and allowed to dry at room temperature. To dissolve the dried dye, 33% acetic acid was dispensed into all tubes. These samples were transferred to a 96-well microplate and measured at 570 nm on a microplate reader (Biotek Synergy H1) in triplicate [25–27].

For the assessment of biofilm formation, the scale is based on the absorbance value of the negative control reported by Chusri et al. [28]. On the other hand, the formula based on the absorbance value of the positive control was used to determine the percentage of biofilm inhibition.

$$\begin{array}{l}{\rm{Biofilm}}\,{\rm{Formation}}\,{\rm{Percentage}}\,\left( \% \right)\, = \\\frac{{[{\rm{OD}}570\left( {{\rm{subtance}}} \right) - {\rm{OD}}570({\rm{negative}}\,{\rm{control}})}}{{{\rm{OD}}570\left( {{\rm{negative}}\,{\rm{control}}} \right)}} \times 100\end{array}$$

$$\begin{array}{l}{\rm{Biofilm}}\,{\rm{Inhibition}}\,{\rm{Percentage}}\,\left( \% \right)\, = \\\frac{{[{\rm{OD}}570({\rm{positive}}\,{\rm{control}}) - {\rm{OD}}570({\rm{substance}})}}{{{\rm{OD}}570\left( {{\rm{positive}}\,{\rm{control}}} \right)}} \times 100\end{array}$$

Additional prepared sample from each subgroup was fixed in 2.5% glutaraldehyde for an hour to examine the formed biofilm of *C. albicans* on the surface of the test samples. The sample was then dehydrated using graded ethanol washes. They were dried at 37 °C in a bacteriological incubator before being mounted and covered in gold. Sample surfaces were analyzed using an SEM apparatus (Hitachi SU5000) with a magnification of 5000×.

SPSS (ver. 29) and GraphPad Prism 8.4.3 programs were used for data analysis. One-way analysis of variance was used in the comparisons between the groups since parametric test assumptions were fulfilled (antibiofilm test). The Kruskal-Wallis test was used because of the non-normal distribution of the surface roughness and contact angle data. The data were submitted to the Mann-Whitney U test for pairwise comparisons. For statistical significance, a *p*-value of less than 0.05 was considered.

## Results

Surface roughness and contact angle results of the test samples are presented in Table 2. The surface roughness values of the 3D-printed test samples were found to be statistically higher than the other groups (*P* < .001). The water contact angle of all test materials was not statistically different from each other (*P* > .001).

**Table 2:** Surface roughness and contact angle values of the test samples

Test groups	Ra (μm) Median (IQR)	Contact angle (◦) Median (IQR)
Cold-polymerized	0.114 (0.06)^a^	73.2 (19.3)^a^
Heat-polymerized	0.123 (0.07)^a^	66.45 (12.55)^a^
CAD/CAM milling	0.119 (0.06)^a^	75.2 (13.13)^a^
3D printing	0.195 (0.06)^b^	69.25 (16.63)^a^
*P* value	**< 0.001**	> 0.001

Interquartile Range (IQR) The different letters in the column indicate a statistically significant difference. (Kruskal-Wallis and Mann-Whitney U Tests)

MIC values of Corega, Carvacrol, and Fluconazole are given in Table 3.

**Table 3:** MIC values of the substances used in the study

Antifungals	MIC (mg/ml)
Corega	20
Carvacrol	0.128
Fluconazole	0.001

(MIC: minimal inhibitory concentration; mg: milligram; ml: milliliter)

The averages and degrees of biofilm produced on denture bases produced by cold-polymerized, heat-polymerized, CAD/CAM milling, and 3D printing methods are shown in Table 4. The average biofilm mass of the heat-polymerized and CAD/CAM milling groups was higher than those of other groups. The degree of biofilm formed on all materials was found to be medium biofilm level according to the biofilm grading method reported by Chusri et al. [28]. (biofilms are classified as non-biofilm if ODtest < ODcontrol, weak biofilm if ODcontrol < ODtest < 2ODcontrol, moderate biofilm if 2ODcontrol < ODtest < 4ODcontrol and strong biofilm if ODtest > 4ODcontrol).

**Table 4:** Averages and degrees of biofilm formed on the test materials

Test materials	x̄_OD_	S_OD_	NC	ODtest/ODcontrol	Biofilm Degree	
Cold-polymerized	2.417	0.066	0.841 ± 0.133	2.87	II	Medium biofilm
Heat-polymerized	0.846	0.029	0.218 ± 0.059	3.88	II	Medium biofilm
CAD/CAM milling	0.648	0.054	0.184 ± 0.061	3.52	II	Medium biofilm
3D printing	2.714	0.046	0.958 ± 0.081	2.83	II	Medium biofilm

(x̄_OD_: Arithmetic mean of optical density, S_OD_: Optical density standard deviation, NC: Negative control)

**Fig. 1:**
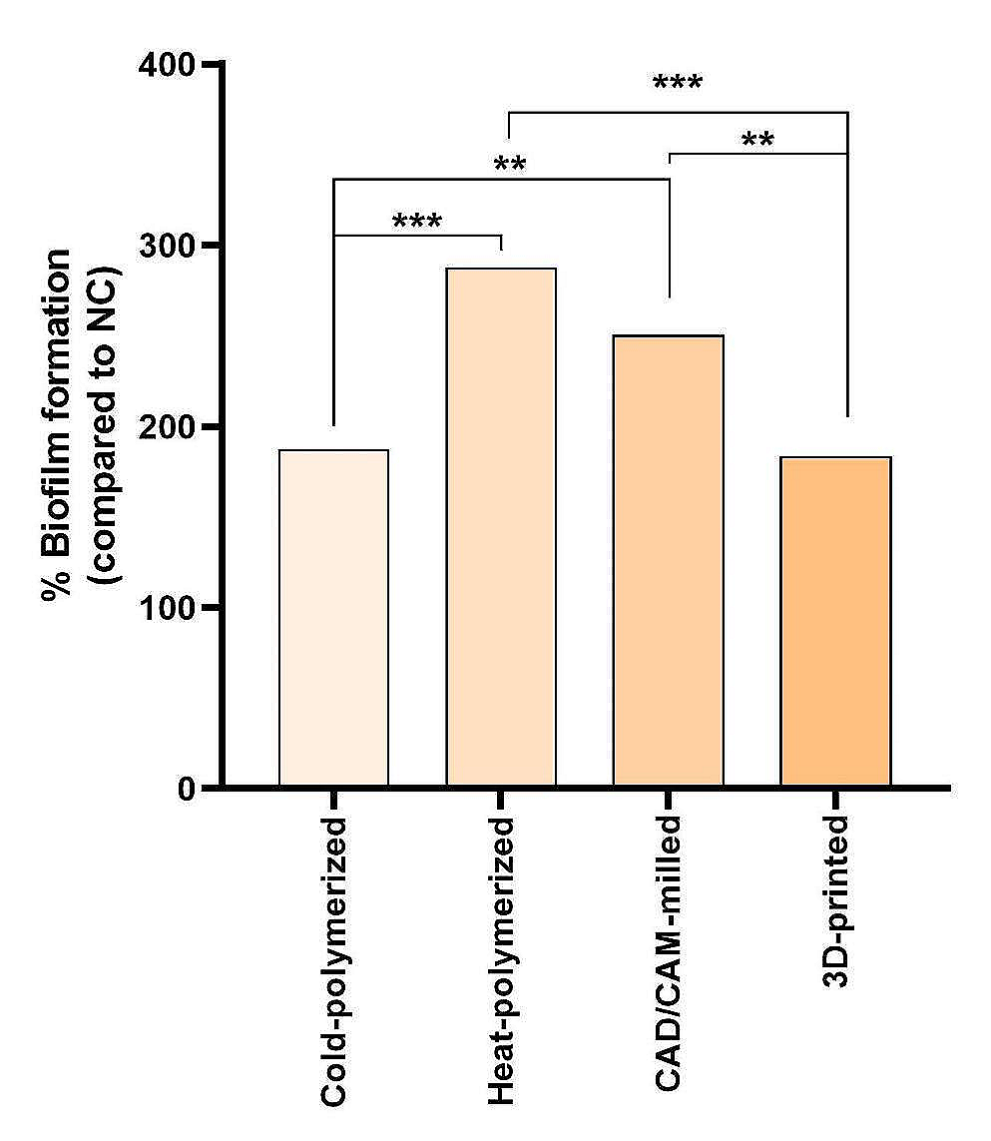
Percentage plot of biofilms produced on the materials compared to the negative control (One-way analysis of variance, **p < .01, ***p < .001)

The quantity of biofilm formed on prosthetic materials acquired by the heat-polymerized and CAD/CAM milling methods showed a significant difference from the amount of biofilm generated on materials obtained by the cold-polymerized and 3D printing method (*P* < .001; *P* < .01) (Fig. 1).

**Fig. 2:**
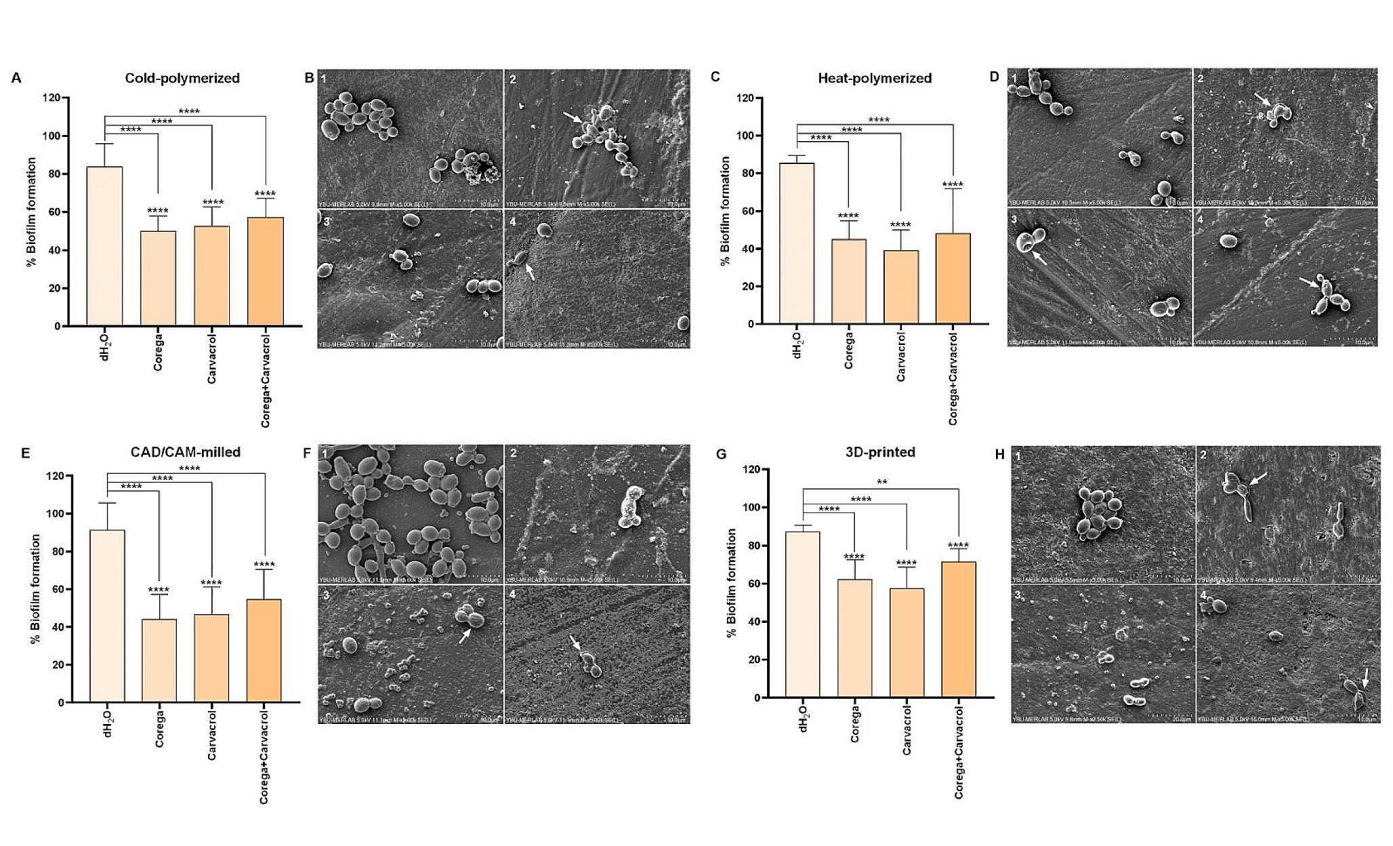
Percent formation plots and electron microscopy images of biofilm formed on test materials produced by cold-polymerized (**A**-**B**), heat-polymerized (**C**-**D**), CAD/CAM milling (**E**-**F**), and 3D printing (**G**-**H**) methods Control (1), Corega treatment (2), Carvacrol treatment (3), Combined of Corega and carvacrol treatment (4) (white arrows indicate volume reduction and morphological changes in Candida cells)

Corega and carvacrol, separately and combined, significantly decreased the amount of biofilm on all surfaces, according to the positive control absorbance value (*P* < .0001) (Fig. 2). There were notable differences between the subgroups of distilled water and other treatments in all test materials (*P* < .0001) (Fig. 2A, C, E, G).

The use of Corega alone or combined with carvacrol was observed and the rate of biofilm inhibition on the surface of the 3D-printed materials was less than that of other groups (*P* < .001; *P* < .05). Carvacrol treatment inhibited the biofilm on the 3D-printed material less than the heat-polymerized resin (*P* < .0001) and CAD/CAM milling materials (*P* < .05) (Table 5).

**Table 5:** Comparison of biofilm inhibition percentages obtained with Corega and carvacrol alone and in combination in four material groups

Grup A	Grup B	Corega				Carvacrol			Corega/ Carvacrol		
		Mean difference (A-B)		Std. error	*P*	Mean difference (A-B)	Std. error	*P*	Mean difference (A-B)	Std. error	*P*
cold-polymerized	heat-polymerized	5.036	2.975		0.333	13.4	3.396	< 0.001*	9.025	4.547	0.202
	CAD/CAM	5.925	2.975		0.199	6.037	3.396	0.291	2.507	4.547	0.946
	3D printed	-12.14	2.975		< 0.001*	-4.764	3.396	0.501	-14.41	4.547	< 0.05*
heat-polymerized	CAD/CAM	0.89	2.975		0.991	-7.364	3.396	0.14	-6.518	4.547	0.482
	3D printed	-17.17	2.975		< 0.0001*	-18.17	3.396	< 0.0001*	-23.44	4.547	< 0.0001*
CAD/CAM	3D printed	-18.06	2.975		< 0.0001*	-10.8	3.396	< 0.05*	-16.92	4.547	< 0.01*

* indicate a statistically significant difference

The treatment of denture cleaning tablet (Corega), carvacrol, or both in the test material in each group decreased *C. albicans* adhesion and colonization compared to those of the control group (Fig. 2B, D, F, H). Although the most intense *C. albicans* adhesion was observed in the CAD/CAM milling control group, colonization of *C. albicans* was exhibited in the other test groups and the highest colonization was detected in the cold-polymerized control group (Fig. 2B, F). In the control groups of 3D printing and CAD/CAM milling, both the hyphal and yeast forms of *C. albicans* were seen, whereas the other groups only saw the yeast form (Fig. 2B, D, F, H). Compared to other test materials, the treatments applied to 3D-printed test material generated less *C. albicans* inhibition (Fig. 2G, H).

Corega treatment applied cold-polymerized, heat-polymerized, and 3D-printed test samples volume reduction was detected in *C. albicans* cells. Morphological changes were detected in the fungal cells for heat-polymerized samples in the carvacrol and combined application, as well as for the CAD/CAM milled test samples in the carvacrol application. In the combined application, a decrease in the volume of fungal cells in the cold-polymerized, CAD/CAM milling, and 3D-printed test samples was observed (Fig. 2).

## Discussion

This study compared the biofilm inhibition effects of denture cleaning tablets, carvacrol, and combined use of *C. albicans* on denture bases produced by digital and conventional methods. The denture base materials’ surface characteristics, such as contact angles and surface roughness, were also assessed. In light of the results obtained from the study, the null hypotheses of the study were partially rejected because the denture cleaning tablet and carvacrol treatment did not show similar antibiofilm effects on the base materials produced by different methods. In addition, the combined use of carvacrol and denture cleaning tablet had no synergistic antibiofilm effect against *C. albicans* compared to alone use. Although there was no significant difference between the contact angle values of the tested materials, the surface roughness values were higher in the 3D-printed test material than in the other groups.

Carvacrol, a monoterpene phenol and one of the main components of oregano (Origanum vulgare) EO, its antifungal action was evaluated against human pathogens such as Candida sp. showing remarkable efficacy. The use of carvacrol has been demonstrated to induce permeability and depolarization of the cell membrane in *C. albicans* cells [29]. Baygar et al. [11] reported that the addition of 10 mL carvacrol to soft lining material significantly reduced biofilm formation and colonization of *C. albicans.*

Considering the increase in antibiotic drug resistance and toxicity, alternative natural agents have been developed [30]. The World Health Organization (WHO) has also launched an active program for alternative therapies based on the use of medicinal plants [31]. Carvacrol demonstrated low toxicity and was effective in treating systemic *C. albicans* infections due to its antifungal and immunomodulatory properties [32]. Herbal treatments are favored over conventional medications because of their abundant natural properties, lower expenses, greater accessibility, and wide safety margin [33]. In the present study, Carvacrol its usability as a denture cleaning agent and its synergistic effect with its addition to the commercial product were investigated.

Surface roughness is a clinically significant factor in predicting of first microbial adherence to dental biomaterials [5, 34]. To prevent biofilm formation on dental prosthetic surfaces, a roughness limit of 0.2 μm has been suggested [4]. Considering the findings of the present study, the surface roughness values of all test groups were found below this threshold value. However, one of the limitations of this study is that the surface roughness after 8 h of immersion was not evaluated again. On the other hand, in a comparison based on material, the test samples generated using the 3D-printed approach had higher surface roughness values than the other groups. This result is consistent with research from Khattar et al. [2] reported that the surface roughness of heat-polymerized resins was lower than that of 3D-printed resin. In addition, although the surface roughness of the test samples produced with 3D-printed was higher than the other test materials, the biofilm mass was found to be lower in the 3D-printed and cold-polymerized groups than in the CAD/CAM milling and heat-polymerized groups. Similarly, they reported that the total biofilm mass was not associated with surface roughness in their study of splint materials produced by 3D printing, CAD/CAM milling, and conventional methods [26]. Biofilm thickness depends on multiple factors beyond merely the number of bacteria forming it. Specifically, environmental conditions like flow, nutrient levels, and temperature are known to impact the structure of the biofilm matrix [35]. This finding suggests that other parameters [interactions between microorganisms, hydrophobicity, surface charge, surface free energy (SFE), structure, and content of the biomaterial] besides surface roughness and surface topography affect microbial adhesion.

Wettability and SFE can also play a role in biofilm adhesion; hydrophobic materials are more likely to form biofilms of *C. albicans* because of non-covalent interactions with the hydrophobic cell wall proteins of *C. albicans* that are expressed by the Csh1p gene. However, the impact of water contact angle has not been demonstrated to be as detrimental as that of surface roughness [19].

In the present study, all test materials showed similar contact angle values. Contrary to these results, Fouda et al. [21] reported that the contact angles of milled resins were significantly lower than those of heat and 3D-printed resins. Alammari [36] observed that the polymerized CAD/CAM acrylic resin denture base materials had low contact angles (about 67 to 70°), which suggests good wettability. Freitas et al. [19] reported that 3D-printed produced resins had a low contact angle (70.82±2.95°) and were hydrophilic, but their high surface roughness (0.317 ± 0.151 μm) might lead to the observation of higher *C. albicans* adhesion. However, Al-Dwairi et al. [37, 38] stated that heat-polymerized resin exhibited a lower contact angle than both milled and 3D-printed resins. Variations in study designs and materials may have led to different findings.

A contact angle value greater than 62° indicates that the material is hydrophobic [39, 40]. On the other hand, it is stated that a water contact angle of less than 90° indicates hydrophilicity, while an angle greater than 90° indicates hydrophobicity [41, 42]. Arslan et al. [43] reported that the water contact angles of CAD/CAM polymers and heat-polymerized PMMA materials were similar. This finding is compatible with our study. The highest values were found in CAD/CAM PMMAs, indicating that CAD-CAM polymers may be more hydrophobic. The greater hydrophobicity may stem from the minimal residual monomer content, as CAD/CAM PMMAs are prepolymerized at high pressure and temperature, which changes the molecular polarity and affects wettability [44].

Biofilms are dynamic communities of microorganisms encased in a secreted polymer matrix, which brings cells into proximity, facilitating the transfer of nutrients, wastes, quorum-sensing signaling molecules, genetic material, and secretions. There are both direct and indirect quantification methods for measuring biofilm formation. One of the direct quantification methods is the colony-forming unit (CFU) method. This method may not be preferable in certain circumstances. Because it is time-consuming and labor-intensive. Sometimes it is difficult to obtain reproducible results and it can take days to repeat a sufficient number of times. There may also be errors due to bacterial aggregation as the biofilm requires suspension. For these reasons, the crystal violet method was preferred in our study as an indirect quantitation method that is easy to perform, reproducible, and allows the evaluation of multiple samples at the same time [45].

In our study, we aimed to inhibit biofilm formation and biofilm formation. Therefore, we preferred the crystal violet method, which is frequently used in microbiological analysis. This method has been used in many studies [34, 46]. Studies using CFU and MTT/XTT methods should also be performed to examine metabolic activity and the status of live and dead microorganisms in biofilm.

Osman et al. [1] evaluated *C. albicans* adhesion and biofilm formation on conventionally manufactured, milled, and 3D-printed denture base resin materials to assess the risk of denture contamination during clinical use. They reported that ranking from the highest to lowest biofilm formation rate was observed in 3D printed, heat polymerized, and milled groups, respectively. They concluded that the production technique affects the surface topography and microbial adhesion of the denture base resin. In the present study, the biofilm mass was highest in the test samples obtained by heat-polymerized and CAD/CAM milling methods, respectively. Although acrylic prostheses produced by 3D-printed and cold-polymerized methods had lower biofilm formation, the degree of biofilm formed on all materials was found to be medium biofilm level according to the biofilm grading method reported by Chusri et al. [28] The reasons for the differences from the aforementioned study can be attributed to the different test methods (Osman et al. [1] XTT method, in the present study crystal violet staining method) and the different material contents used. In addition, the test samples’ geometric shapes and the use of polishing and finishing techniques could cause different results (Osman et al. [1] unpolished, in the present study polishing). Moreover, the incubation period may also have caused the different results.

The final product’s quality and surface roughness in the additive manufacturing process is determined by the printing parameters chosen (such as the printing angle, layer thickness, and support structure orientation), the type of printer used, the printing technique, and the post-processing procedure [34]. All of these factors affect the degree of polymerization, physical properties, surface roughness, and microbiological characteristics of the final material [1]. The literature presents contradictory findings about the effects of varying printing orientations (0°, 45°, 90°) on the mechanical properties, microbiological reaction, and physical attributes of 3D-printed denture base resin material. A 90° construction orientation was suggested by Shim et al. [47] despite its relatively low flexural strength, due to its high precision, low roughness, and decreased *C. albicans* attachment. Li et al. [34], however, discovered no correlation between *C. albicans* adhesion on 3D-printed denture base resins and the additive manufacturing techniques [stereolithography (SLA) and digital light processing (DLP)] or print orientation (0°, 45°, 90°). Lee et al. [5] reported that 0° build angle exhibited the lowest surface roughness independent of viscosity and 50 μm layer thickness caused the lowest *C. albicans* attachment. In the current study, liquid crystal display (LCD) fabrication technology was used with a printing angle of 0° and a layer thickness of 50 μm. Carvacrol treatment caused the highest biofilm inhibition for 3D-printed test samples. However, due to different study designs, these results could not be compared directly with previous studies. Further studies using natural denture cleaning products on 3D-printed denture bases produced with different angles and manufacturing techniques are required.

According to Meirowitz et al. [3], additively produced samples showed high candida adhesion, while subtractively produced samples showed low candida adhesion. Also, heat- and cold-polymerized samples showed a value between the additive and subtractive groups. However, the authors found that mucin adsorption, not the material’s surface roughness, was associated with higher fungal adhesion on the printed surface. The different results from the present study may be attributed to mucin adsorption. Microbiological behavior may be related to the chemical composition.

The reason for the lower antibiofilm effect of carvacrol and Corega treatments in test samples produced with 3D printing may be related to the rougher surface of this material. These results suggest that *C. albicans* cannot be inhibited alone by prosthesis cleaning agents; instead, a combined approach involving mechanical cleaning and immersion in cleaning agents may need to be used for this material.

Niu et al. [48] reported in a study that Carvacrol increased the membrane permeability of yeast cells. In another study, the effect of carvacrol on ergosterol, the cell membrane sterol of candidae was examined. According to this study stated that Carvacrol inhibited ergosterol synthesis and caused a change in cell membrane permeability [49]. Carvacrol may function as an endoplasmic reticulum (ER) stressor, according to a different study on *C. albicans*. Unfolded protein reaction results from disruption of the ER or disruption of ER organization in carvacrol-treated cells [50]. Considering the SEM findings from the present study, it can be inferred that the decrease in the volume of yeast cells after corega and combined application may as a result of the cell membrane disrupting the membrane integrity.

The more infected form of the candida (infectious), hyphae formation, and phenotypic transition play a role in the virulence of the fungus [51]. The SEM findings of the present study suggest that the candida growing on the CAD/CAM material may have acquired a more virulent characteristic than the other materials since it showed hyphae formation.

The limitations of this study include the fact that the prepared samples were disk and polished and could not fully simulate the clinical situation due to the complex production of the denture base, the use of only *C. albicans* species due to the presence of numerous other species in the biofilm layer in the oral environment, and the use of a single orientation angle and production technique in the preparation of 3D-printed denture test samples. Additionally, more studies using different natural cleaning products to prevent DS are needed.

## Conclusions

1. The surface roughness values of the digitally and conventionally produced test samples were within the clinically acceptable threshold (below 0.2 μm).
2. All test materials showed similar water contact angle values.
3. The treatment of carvacrol and denture cleaning tablets to denture bases produced by different methods affects biofilm inhibition against *C. albicans.*
4. Carvacrol may be used as one of the disinfectant agents for denture cleaning due to its biofilm inhibition property. However, carvacrol combined use with a cleaning tablet did not produce a synergistic effect.

## Data Availability

All data on biofilm inhibition of denture cleaning tablets and carvacrol on denture bases supporting the findings of this study are included in this paper and are not openly available but can be obtained from the corresponding author upon request.
